# Supplementary material for: Resolving the full spectrum of human genome variation using Linked-Reads
Source: Genome Res. 2019 Apr;29(4):635–45. doi: 10.1101/gr.234443.118 (PMC6442396; doi:10.1101/gr.234443.118)
Supplement: Supplemental Material [file supp_gr.234443.118_Supplemental_longranger-2.2.zip › longranger-2.2.tar.bz2!/lr2.2-public/lib/stan/cmdstan/stan/doc/api/html/multi__gp__cholesky_8hpp_source.html]

Stan: src/stan/prob/distributions/multivariate/continuous/multi\_gp\_cholesky.hpp Source File


|  |  |
| --- | --- |
|  | Stan  2.5.0  probability, sampling & optimization |


- Main Page
- Related Pages
- Namespaces
- Classes
- Files

- File List
- File Members

All Classes Namespaces Files Functions Variables Typedefs Enumerations Enumerator Friends Macros Pages

- src
- stan
- prob
- distributions
- multivariate
- continuous

multi\_gp\_cholesky.hpp

Go to the documentation of this file.

1 #ifndef STAN\_\_PROB\_\_DISTRIBUTIONS\_\_MULTIVARIATE\_\_CONTINUOUS\_\_MULTI\_GP\_CHOLESKY\_HPP

2 #define STAN\_\_PROB\_\_DISTRIBUTIONS\_\_MULTIVARIATE\_\_CONTINUOUS\_\_MULTI\_GP\_CHOLESKY\_HPP

3

4 #include <stan/math/matrix\_error\_handling.hpp>

5 #include <stan/math/error\_handling.hpp>

6 #include <stan/math/error\_handling/dom\_err.hpp>

7 #include <stan/prob/constants.hpp>

8 #include <stan/prob/traits.hpp>

9 #include <stan/agrad/rev.hpp>

10 #include <stan/meta/traits.hpp>

11 #include <stan/agrad/rev/matrix.hpp>

12 #include <stan/math/matrix/row.hpp>

13 #include <stan/math/matrix/dot\_self.hpp>

14 #include <stan/math/matrix/log.hpp>

15 #include <stan/math/matrix/multiply.hpp>

16 #include <stan/math/matrix/sum.hpp>

17 #include <stan/math/matrix/mdivide\_left\_tri\_low.hpp>

18

19 namespace stan {

20  namespace prob {

21  // MultiGPCholesky(y|L,w) [y.rows() = w.size(), y.cols() = Sigma.rows();

22  // Sigma symmetric, non-negative, definite]

42  template <bool propto,

43  typename T\_y, typename T\_covar, typename T\_w>

44  typename boost::math::tools::promote\_args<T\_y,T\_covar,T\_w>::type

45  multi\_gp\_cholesky\_log(const Eigen::Matrix<T\_y,Eigen::Dynamic,Eigen::Dynamic>& y,

46  const Eigen::Matrix<T\_covar,Eigen::Dynamic,Eigen::Dynamic>& L,

47  const Eigen::Matrix<T\_w,Eigen::Dynamic,1>& w) {

48  static const char\* function = "stan::prob::multi\_gp\_cholesky\_log(%1%)";

49  typedef typename boost::math::tools::promote\_args<T\_y,T\_covar,T\_w>::type T\_lp;

50  T\_lp lp(0.0);

51

52  using stan::math::mdivide\_left\_tri\_low;

53  using stan::math::dot\_self;

54  using stan::math::sum;

55  using stan::math::log;

56

57  using stan::math::check\_size\_match;

58  using stan::math::check\_finite;

59  using stan::math::check\_positive;

60

61  check\_size\_match(function,

62  y.rows(), "Size of random variable (rows y)",

63  w.size(), "Size of kernel scales (w)",

64  &lp);

65  check\_size\_match(function,

66  y.cols(), "Size of random variable",

67  L.rows(), "rows of covariance parameter",

68  &lp);

69  check\_finite(function, w, "Kernel scales", &lp);

70  check\_positive(function, w, "Kernel scales", &lp);

71  check\_finite(function, y, "Random variable", &lp);

72

73  if (y.rows() == 0)

74  return lp;

75

76  if (include\_summand<propto>::value) {

77  lp += NEG\_LOG\_SQRT\_TWO\_PI \* y.rows() \* y.cols();

78  }

79

80  if (include\_summand<propto,T\_covar>::value) {

81  lp -= L.diagonal().array().log().sum() \* y.rows();

82  }

83

84  if (include\_summand<propto,T\_w>::value) {

85  lp += 0.5 \* y.cols() \* sum(log(w));

86  }

87

88  if (include\_summand<propto,T\_y,T\_w,T\_covar>::value) {

89  T\_lp sum\_lp\_vec(0.0);

90  for (int i = 0; i < y.rows(); i++) {

91  Eigen::Matrix<T\_y, Eigen::Dynamic, 1> y\_row( y.row(i) );

92  Eigen::Matrix<typename boost::math::tools::promote\_args<T\_y,T\_covar>::type,

93  Eigen::Dynamic, 1>

94  half(mdivide\_left\_tri\_low(L,y\_row));

95  sum\_lp\_vec += w(i) \* dot\_self(half);

96  }

97  lp -= 0.5\*sum\_lp\_vec;

98  }

99

100  return lp;

101  }

102

103  template <typename T\_y, typename T\_covar, typename T\_w>

104  inline

105  typename boost::math::tools::promote\_args<T\_y,T\_covar,T\_w>::type

106  multi\_gp\_cholesky\_log(const Eigen::Matrix<T\_y,Eigen::Dynamic,Eigen::Dynamic>& y,

107  const Eigen::Matrix<T\_covar,Eigen::Dynamic,Eigen::Dynamic>& L,

108  const Eigen::Matrix<T\_w,Eigen::Dynamic,1>& w) {

109  return multi\_gp\_cholesky\_log<false>(y,L,w);

110  }

111  }

112 }

113

114 #endif

matrix.hpp

mdivide\_left\_tri\_low.hpp

dom\_err.hpp

stan::math::mdivide\_left\_tri\_low

Eigen::Matrix< typename boost::math::tools::promote\_args< T1, T2 >::type, R1, C2 > mdivide\_left\_tri\_low(const Eigen::Matrix< T1, R1, C1 > &A, const Eigen::Matrix< T2, R2, C2 > &b)

**Definition:** mdivide\_left\_tri\_low.hpp:16

stan::agrad::dot\_self

fvar< T > dot\_self(const Eigen::Matrix< fvar< T >, R, C > &v)

**Definition:** dot\_self.hpp:16

stan::math::dot\_self

double dot\_self(const std::vector< double > &x)

**Definition:** dot\_self.hpp:11

traits.hpp

stan::prob::multi\_gp\_cholesky\_log

boost::math::tools::promote\_args< T\_y, T\_covar, T\_w >::type multi\_gp\_cholesky\_log(const Eigen::Matrix< T\_y, Eigen::Dynamic, Eigen::Dynamic > &y, const Eigen::Matrix< T\_covar, Eigen::Dynamic, Eigen::Dynamic > &L, const Eigen::Matrix< T\_w, Eigen::Dynamic, 1 > &w)

The log of a multivariate Gaussian Process for the given y, w, and a Cholesky factor L of the kernel ...

**Definition:** multi\_gp\_cholesky.hpp:45

stan::math::check\_size\_match

bool check\_size\_match(const char \*function, T\_size1 i, const char \*name\_i, T\_size2 j, const char \*name\_j, T\_result \*result)

**Definition:** check\_size\_match.hpp:13

error\_handling.hpp

stan::math::check\_finite

bool check\_finite(const char \*function, const T\_y &y, const char \*name, T\_result \*result)

Checks if the variable y is finite.

**Definition:** check\_finite.hpp:52

log.hpp

matrix\_error\_handling.hpp

constants.hpp

stan::agrad::sum

fvar< T > sum(const Eigen::Matrix< fvar< T >, R, C > &m)

**Definition:** sum.hpp:14

traits.hpp

dot\_self.hpp

stan::prob::include\_summand

Template metaprogram to calculate whether a summand needs to be included in a proportional (log) prob...

**Definition:** traits.hpp:35

rev.hpp

stan::math::check\_positive

bool check\_positive(const char \*function, const T\_y &y, const char \*name, T\_result \*result)

**Definition:** check\_positive.hpp:54

stan::math::log

Eigen::Matrix< T, Rows, Cols > log(const Eigen::Matrix< T, Rows, Cols > &m)

Return the element-wise logarithm of the matrix or vector.

**Definition:** log.hpp:16

row.hpp

stan::math::sum

double sum(std::vector< double > &x)

**Definition:** sum.hpp:10

stan::agrad::mdivide\_left\_tri\_low

Eigen::Matrix< fvar< T >, R1, C1 > mdivide\_left\_tri\_low(const Eigen::Matrix< fvar< T >, R1, C1 > &A, const Eigen::Matrix< fvar< T >, R2, C2 > &b)

**Definition:** mdivide\_left\_tri\_low.hpp:22

sum.hpp

stan::agrad::log

fvar< T > log(const fvar< T > &x)

**Definition:** log.hpp:15

multiply.hpp


---

[ Stan Home Page ]

*© 2011–2014,
Stan Development Team.*
